# DNA methylation, environmental exposures and early embryo development

**DOI:** 10.21451/1984-3143-AR2019-0062

**Published:** 2019-10-23

**Authors:** Mélanie Breton-Larrivée, Elizabeth Elder, Serge McGraw

**Affiliations:** 1 Department of Biochemistry and Molecular Medicine, Université de Montréal, Research Center of the CHU Sainte-Justine. Montreal, Canada.; 2 Department of Obstetrics & Gynecology, Université de Montréal, Research Center of the CHU Sainte-Justine, Montréal, Canada.

**Keywords:** epigenetics, DNA methylation, pre-implantation embryos, prenatal exposures, developmental programming

## Abstract

The first crucial step in the developmental program occurs during pre-implantation, the time after the oocyte has been fertilized and before the embryo implants in the uterus. This period represents a vulnerable window as the epigenome undergoes dynamic changes in DNA methylation profiles. Alterations in the early embryonic reprogramming wave can impair DNA methylation patterns and induce permanent changes to the developmental program, leading to the onset of adverse health outcomes in offspring. Although there is an increasing body of evidence indicating that harmful exposures during pre-implantation embryo development can trigger lasting epigenetic alterations in offspring, the mechanisms are still not fully understood. Since physiological or pathological changes in DNA methylation can occur as a response to environmental cues, proper environmental milieu plays a critical role in the success of embryonic development. In this review, we depict the mechanisms behind the embryonic epigenetic reprogramming of DNA methylation and highlight how maternal environmental stressors (e.g., alcohol, heat stress, nutrient availability) during pre-implantation and assisted reproductive technology procedures affect development and DNA methylation marks.

## Introduction

The rapidly emerging field of epigenetics studies genome modifications that regulate gene expression without altering the content of the genetic sequence. DNA and histones —*the structural proteins of the chromatin*— can possess a layer of reversible epigenetic modifications that contribute to how genes are expressed and how they interact within a cell. Epigenetic modifications are chemical tags, such as phosphate, methyl and acetyl groups, affixed to the histone proteins and DNA by a highly dynamic and synergic network of nuclear enzymes that modulate chromatin availability thereby regulating gene expression (Jenuwein and Allis, 2001; Gibney and Nolan, 2010). The epigenome is of utmost importance and comprehensively susceptible to environmental factors, (Marsit, 2015; Legault *et al*., 2018; Norouzitallab *et al*., 2019) particularly during the early stages of embryo development as its epigenetic regulation is concomitant with proper cell fate determination (Morey *et al*., 2015; Ohbo and Tomizawa, 2015; Vougiouklakis *et al*., 2017). The most notable epigenetic mechanism during mammalian pre-implantation is the epigenetic reprogramming of DNA methylation that triggers embryonic genome activation, a pivotal step for proper embryo development. While these processes are similar between species, they differ in regards to the rate and timing of events and sex-specific variations. Although many studies have shown the highly significant physiological roles of the epigenome in mammalian development, it is still considerably misunderstood and insufficiently studied during pre-implantation, partially due to technological limitations as a consequence of the very small number of cells and the short duration of this stage of development. This review will depict the mechanisms behind the embryonic epigenetic reprogramming of DNA methylation and will assess the epigenetic consequences of various assisted reproductive technology (ART) procedures as well as how environmental stressors during pre-implantation will affect short-term and long-term development, focussing specifically on the maternal environment.

### DNA Methylation

DNA methylation is the most widely understood epigenetic modification as a mechanism for gene expression mediation and is involved in many key physiological processes such as genomic imprinting, transposable elements silencing, X-chromosome inactivation and aging (Bird, 2002; Smith and Meissner, 2013). In mammals, DNA methylation occurs mainly on the cytosines of cytosine-guanine dinucleotides known as CpG sites (CpGs) (Razin and Cedar, 1991; Weber and Schubeler, 2007), though non-CpG (i.e. CpA, CpT, CpC) methylation can also be found at specific stages of cellular development, primarily in stem cells and brain tissues (Lister *et al*., 2013; Patil *et al*., 2014). The enzymes directly responsible for the methylation of DNA are DNA methyltransferases (DNMTs). DNMT3A and DNMT3B add *de novo* methylation thus they have been identified to be involved in the establishment of methylation patterns required for cell lineage determination during development (Okano *et al*., 1999; Li, 2002). For optimal activity, DNMT3A and DNMT3B require the accessory protein DNMT3L (DNA methyltransferase-like), a protein similar to DNMTs but lacking methyltransferase activity (Suetake *et al*., 2004). In contrast, DNMT1 carries out heritable DNA methylation pattern maintenance during cellular division due to its preference for substrates with hemi-methylated CpGs, *i.e.* only one methylated DNA strand, which would naturally occur during semi-conservative DNA replication (Leonhardt *et al*., 1992; Lei *et al*., 1996; Pradhan *et al*., 1999). Although DNA methylation patterns are heritable from cell to cell, it remains strikingly dynamic in nature. Physiological or pathological changes in DNA methylation can occur as a response to environmental cues, therefore demethylation is conjointly a greatly relevant process. Contrary to enzyme-mediated methylation, demethylation can occur passively or actively (Kishikawa *et al*., 2003). Passive genome demethylation is replication-dependant, caused by DNMT1 reduced activity resulting in an unspecific progressive dilution of DNA methylation over multiple consecutive cell divisions (Kagiwada *et al*., 2013; Wu and Zhang, 2014). Conversely, active demethylation is specific and is catalytically directed by ten-eleven translocation enzymes (TET1, TET2, TET3) (Tahiliani *et al*., 2009; Ito *et al*., 2010). The addition and erasure of DNA methylation and other epigenetic marks are the driving forces behind embryo development, as they dictate how, when and at what level genes are expressed.

CpGs are present all across the mammalian genome but their methylation will bear different consequences depending upon their location (e.g. promoter regions, gene bodies, enhancers) and upon their level of enrichment. CpG methylation located in gene bodies has been shown to promote high levels of gene expression whereas when located in promoter regions, it is associated with transcriptional silencing, which coordinates cellular differentiation (Goll and Bestor, 2005; Jones, 2012). In mammalian genomes, promoters comprised of highly CpG dense sequences, known as CpG islands (CGIs), control approximately 60-80% of genes depending on the species (Antequera and Bird, 1993; Saxonov *et al*., 2006). Methylation of CGIs of a promoter accompanied with repressive histone modifications (H3K9me3, H3K27me3) induces nucleosome compaction and prevents transcription factor (TF) binding, causing the repression of gene transcription. On the other hand, the promoter regions of transcribed genes have CGIs devoid of methylation along with active histone modifications (H3K4me2/3, H3K9ac) (Barski *et al*., 2007; Koch *et al*., 2007; Henikoff and Shilatifard, 2011; Severin *et al*., 2011), thus ensuring the open chromatin configuration that allows for TF binding and gene activation. However, recent studies have started to refute this general rule suggesting that, in some cases, the loss of methylation can be a consequence of TF binding as opposed to the cause of action, leading some to believe that gene activation may not always be methylation driven (Zhu *et al*., 2016a; Pacis *et al*., 2019).

A particularly important role of DNA methylation in mammalian development is genomic imprinting. A small cohort of genes called imprinted genes possesses germline differentially methylated regions (gDMRs). gDMRs acquire monoallelic genomic methylation in a parent-of-origin manner causing only one allele to be expressed (Reik *et al*., 2001; Ferguson-Smith, 2011). A more specific type of gDMR is imprinting control regions (ICRs) that are directly implicated in the binding of TFs and regulate the expression of multiple imprinted genes at a time (e.g., *H19* and *Igf2; Insulin-Like Growth Factor 2*) (Thorvaldsen *et al*., 1998; Fitzpatrick *et al*., 2002; Ideraabdullah *et al*., 2008). These genomic imprints are determined prior to fertilization in growing diplotene oocytes and in perinatal prospermatogonia and must be maintained throughout the entire lifespan of the new generation (Stoger *et al*., 1993; Kono *et al*., 1996; Davis *et al*., 2000; Ueda *et al*., 2000).

### Embryonic Epigenetic Reprogramming

In early embryogenesis, the embryo undergoes a reprogramming wave of DNA methylation during which the global methylation profiles, with the exception of gDMRs, are remodeled. Shortly after fertilization, the zygotic genome remains separated into two distinct paternal and maternal pronuclei which must sustain extensive global demethylation to erase the germ cell-specific methylation profiles and implement totipotency prior to implantation of the embryo (Seisenberger *et al*., 2013). The demethylation mechanisms are known to be distinct for both pronuclei, but have not been fully characterized thus far. Another perplexing part of the reprogramming process is the maintenance of gDMR methylation patterns as it is a major requirement for normal mammalian development. In fact, the loss of genomic imprints during embryo development causes permanent damage to cellular functions since the embryo is unable to restore them (Howell *et al*., 1998; Howell *et al*., 2001; McGraw *et al*., 2013; McGraw *et al*., 2015). Since only one allele is inherently active, imprinted gene expression is hypersensitive to changes in regulation, which can cause dramatic effects on development as many imprinted genes have growth regulatory functions (Plasschaert and Bartolomei, 2014). Many studies in mice have demonstrated the prevalent involvement of DNMT1 variants (DNMT1o; DNMT1-oocyte, DNMT1s; somatic) in the maintenance of genomic imprints throughout embryonic epigenetic reprograming (Bostick *et al*., 2007; Arita *et al*., 2008; Avvakumov *et al*., 2008). How DNMT1 specifically recognizes and maintains gDMRs but does not maintain global methylation remains mostly unclear. However, *Dnmt1^-/-^* mice are embryonic lethal as the absence of *Dnmt1* causes the exhaustive loss of genomic imprints and does not allow for *de novo* methylation to be properly maintained during remethylation (Li *et al*., 1992). Moreover, we observed that the loss of *Dnmt1o* caused sex-specific placental defects in female embryos as well as perturbed imprinted X-inactivation (McGraw *et al*., 2013). These data highlight how a brief perturbation in the DNA methylation maintenance process of early stage embryoscan influence development, and further emphasize the importance of studying the impact of the maternal environment and sex-specific alterations during this critical period.

**Figure 1:**
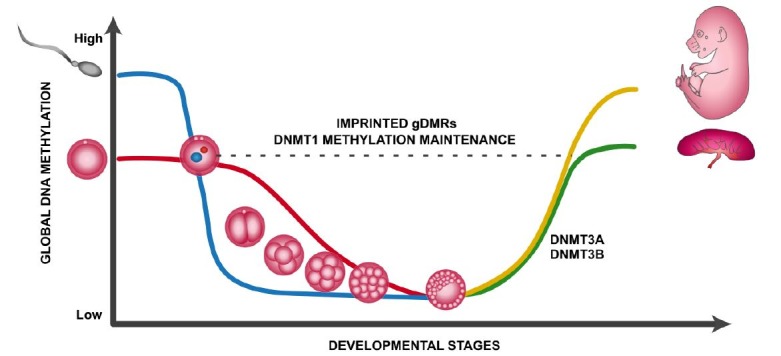
Global DNA demethylation and remethylation during the epigenetic reprogramming of early embryogenesis in mice. Soon after fertilization, the zygotic paternal and maternal pronuclei undergo global demethylation during the pre-implantation stages, except for gDMRs which are maintained via DNMT1 activity. The paternal genome (blue) is initially actively demethylated by the TET3 enzyme followed by passive demethylation, whereas the maternal genome (red) demethylation is solely passive due to DNMT1 inactivity, hence the sharper demethylation slope for the paternal curve. After implantation, the blastocyst acquires *de novo* methylation patterns catalyzed by DNMT3A and DNMT3B to establish the embryonic and placental programs imperative for development initiation.

The loss of global DNA methylation during reprogramming initiates the embryonic genome activation, vital for proper development. The thoroughly timed expression of genes in embryonic genome activation is controlled by chromatin structural changes. A schematic representation of the mouse embryonic epigenetic reprogramming of DNA methylation is depicted in Figure 1, showing the dynamics of DNA methylation from the fertilization of the zygote to the maturation of embryonic and placental tissues. After global demethylation, the inner cell mass and the trophoblast gain *de novo* methylation catalyzed by DNMT3A and DNMT3B to implement the epigenetic patterns for the development of the embryo and the placenta (Red-Horse *et al*., 2004; Marikawa and Alarcon, 2009). Studies conducted in mice have indicated separate specific phenotypes in *Dnmt3a^-/-^* versus *Dnmt3b^-/-^*. *Dnmt3a^-/-^* mice make it to term, albeit severely runted and die shortly thereafter, whereas *Dnmt3b^-/-^* are embryonic lethal (Niakan *et al*., 2012). The altered expression of these key enzymes is unmistakably symptomatic of epigenetic developmental disturbances, but it is becoming more and more evident that the regulation of the epigenome is also staggeringly sensitive to embryonic environment, particularly throughout the pre-implantation period.

## Early embryonic environment and impact on epigenetic reprogramming events

A considerable amount of evidence has begun to show how epigenetic programming is susceptible to early embryonic environment, such as nutrient availability and toxin exposures, prior to implantation of the blastocyst. The dynamics of the embryonic wave of DNA methylation – proper erasure and *de novo* methylation or methylation maintenance – that is crucial to trigger the developmental program can become disturbed in response to these environmental cues leading to changes in gene expression and growth defects. Many have investigated the effects of the environment on embryo development using mainly *in vitro* models. However, the direct epigenetic impacts of the embryonic environment and the lasting effects on long-term development have been poorly studied, due to technological barriers and limited number of cells during pre-implantation stages. When studying early embryonic development exposures, one must be cautious when comparing *in utero* and *in vitro* models being that *in vitro* culture technologies have yet to accurately reproduce the maternal tract conditions. We have thus divided the following section describing the effects of environmental factors during early embryonic development in two parts: firstly, the influence of *in vitro* reproductive technologies of mammalian embryos, and secondly, the intrauterine exposures during pregnancy.

### Assisted Reproductive Technology (ART)

ART is an umbrella term used to describe the assortment of medical procedures and approaches (e.g., superovulation, *in vitro* fertilization (IVF), intracytoplasmic sperm injection (ICSI), embryo *in vitro* culture (IVC)) that can be performed to achievepregnancy. Such procedures are now an integral part of human infertility treatment, as the number of children born by ART is estimated at more than 8 million worldwide (Weinerman, 2018). ART also plays a major role in animal reproduction to increase reproductive efficiency and genetic improvement in livestock, as well as to conserve endangered species. The outcomes of human pregnancies produced by ART have undergone intense scrutiny and while most children conceived using ART are healthy, these procedures have been associated with an increased risk of preeclampsia, intrauterine growth restriction, birth defects (Qin *et al*., 2016; Zhu *et al*., 2016b; Choux *et al*., 2018; Choufani *et al*., 2019) and imprinting disorders (Market-Velker *et al*., 2010; White *et al*., 2015). Although various studies have shown that ART may lead to epigenetic perturbations (El Hajj and Haaf, 2013; Urrego *et al*., 2014; Duranthon and Chavatte-Palmer, 2018), the etiology associated with ART and increased risk of perinatal complications is still poorly understood. However, the dynamic epigenome reprogramming during germ cell development and the pre-implantation period, especially of DNA methylation patterns, are processes that are prone to being affected by approaches used in ART and could provide biological plausibility.

### ART procedures

Although it is unclear which ART procedure has the greatest influence, we know that the dramatic changes in embryo environment can induce long-term effects on the epigenome. In ovarian superstimulation, various studies suggest that the acquisition of imprinting patterns in the oocyte might be perturbed and lead to abnormal allelic expression in later embryo and placenta development (reviewed in Anckaert *et al*., 2013; McGraw and Trasler, 2013). However, studies show that superovulation treatments do not alter normal imprinted methylation acquisition in oocytes, but rather disrupt maternal-effect gene products that are required during pre-implantation for imprint maintenance (Denomme *et al*., 2011; Uysal *et al*., 2018). We also showed that some of these induced errors of imprinted gene expression (*H19*, *Igf2*) present in mid-gestation mouse placenta are no longer apparent at the end of the gestation (Fortier *et al*., 2008; Fortier *et al*., 2014). This suggests that even though superovulation produces abnormal oocytes that initiate altered expression of imprinted genes in embryos, compensatory mechanisms regulating imprinted gene networks are able to restore proper levels of gene expression during development. Although, as highlighted across the literature, the alterations in DNA methylation following ART procedures are not always striking and vary between studies (reviewed in Berntsen *et al*., 2019), in part because of distinctions in treatments used. It was recently reported that human placenta, but not cord blood, from IVF/ICSI showed decreased DNA methylation levels for imprinted loci *H19/IGF2* and *KCNQ1OT1*, as well as for specific repetitive elements (Choux *et al*., 2018), whereas in another recent study, no obvious overall differences in genome-wide DNA methylation differences in placental tissues were associated with ART (Choufani *et al*., 2019). Yet, a subset of ART pregnancies associated with ICSI showed marked decrease in placental DNA methylation levels at imprinted loci (*GNAS*, *SGCE*, *KCNQ1OT1* and *NNAT*). Not only do these studies reveal that ICSI generates distinct DNA methylation alterations in specific tissues compared to controls as opposed to less invasive ART procedures, they highlight the importance of carefully pairing and comparing equivalent ART procedures when designing epigenetic studies.

Another ART procedure that is routine practice in commercial and clinical settings is cryopreservation of oocytes and embryos. Flash-freezing cryopreservation protocols (i.e., vitrification) have been linked to epigenetic alterations. Selective loss of DNA methylation of imprinted loci was observed in blastocysts subsequent to fertilization of vitrified bovine and mouse oocytes (Chen, Zhang *et al*., 2016; Cheng *et al*., 2014), whereas others found no effect on DNA methylation levels at the *H19/IGF2* ICR loci at embryonic day 3 in human ICSI blastocysts following vitrification (Derakhshan-Horeh *et al*., 2016). When vitrification of mouse embryo at E2.5 (8-cell stage) was paired with IVC, transferred embryos revealed increased levels of global DNA methylation in both E9.5 fetus and placenta compared to IVC, but interestingly were similar to naturally mated derived samples (Ma *et al*., 2019). The long-term effect of vitrification was further observed in the fetus with increased DNA methylation levels at the imprinted *KvDMR1* loci and significant gene expression increase of *Dnmt1* and *Dnmt3b* compared to the IVC and natural mating groups. Together, the body of work on vitrification suggests that such exposures could influence the epigenome and lead to abnormal expression of imprinted genes. However, it is difficult to make any definitive conclusions regarding the influence of vitrification as most of these studies only assessed a limited number of loci for DNA methylation analyses, which are mostly restricted to imprinted genes, and did not investigate the long-term impact on postnatal development.

### ART culture environment

As previously mentioned, although the vast majority of ART-conceived offspring are healthy, they have a higher frequency of birth defects suggesting epigenetic costs. A large body of research now supports that the *in vitro* culture environment has both long-lasting and significant repercussions on DNA methylation reprogramming events and embryonic development, but the exact mechanisms remain unclear. In humans, an increased prevalence of Beckwith-Wiedemann syndrome has been associated with ART procedures. This overgrowth disorder has similar adverse phenotypes and epigenetic profiles (e.g., loss of imprinting) as the large offspring syndrome in ruminants, (Chen *et al*., 2015) for which the incidence has been linked to the presence of serum in the culture media. (Young *et al*., 1998; Chen *et al*., 2013). As such,the ART field has mostly limited the use of serum and has designed various serum-free and chemically defined media for livestock, mice, and humans.

Various commercial and custom culture systems exist but are not as complex and dynamic as the oviduct fluid. They may present a lack or excess of different key factors and metabolites when compared to the maternal reproductive environment (Morbeck *et al*., 2014a; Morbeck *et al*., 2014b; Morbeck *et al*., 2017). A number of different studies have investigated the impact of culture systems on epigenetic profiles in humans and other animals, but a direct correlation between results is challenging as additional associated parameters (e.g., culture conditions, protocols, ART-procedures) may introduce a range of confounding and unpredictable variables. To circumvent these effects, Market-Velker *et al*. (2010) undertook a direct side-by-side comparison between naturally mated mouse embryos cultured from the 2-cell stage to the blastocyst stage in commercial systems and *in vivo*-derived blastocyst. They uncovered that all commercial media compromised the early embryo’s proficiency in maintaining genomic imprinting profiles of *H19*, *Peg3,* and *Snrpn* to a variable extent. Although some media systems appeared to be more suitable for maintaining DNA methylation levels on these imprinted loci, we cannot know how the rest of the genome behaves under these conditions because of the narrow epigenetic analyses that were performed. Interestingly, a recent study tested the addition of natural reproductive fluids in the culture system to safeguard the embryo’s epigenome. They showed that by using natural reproductive fluids they could produce IVF-blastocysts with reduced morphological, epigenetic and transcriptomic anomalies when compared to porcine blastocysts produced from unsupplemented IVF protocols (Canovas *et al*., 2017). Furthermore, by using both whole-genome DNA methylation and RNA-seq approaches of single blastocysts, they were able to demonstrate that the addition of oviductal tract fluid compensated for the lack of specific factors in standard culture medium required for proper development. Since this strategy has been successful so far in improving the ART procedures in mice, humans and other livestock animals, it shows great potential for rescuing troubled early embryo development and future negative impacts in offspring.

### Maternal and environmental influences

It is now well established that the maternal environment (e.g., nutrition, stress, toxicants) can create an adverse *in utero* milieu that affects the fetal developmental program and increase disease susceptibility in adulthood (aka. Developmental Origins of Health and Disease; DoHaD hypothesis). Since the *all-or-none* phenomenon once presumed that exposure that occurs on early stage embryos results in either death or in no adverse outcome, little research on the impact of harmful maternal environment on pre-implantation embryos was done in the past. However, this once pervasive tenet is now being revisited as several studies demonstrate that the direct contact of pre-implantation embryo with the cells of the mother’s reproductive tract can influence future development via interference with epigenetic mechanisms (Adam, 2012). Here, we will underline how adverse *in uterine* conditions triggered by the maternal environment (alcohol, heat stress) can have deleterious effects on the early embryonic epigenome.

### Adverse stressors

Alcohol has teratogenic and neurotoxic effects on numerous potential mechanisms such as folate metabolism and DNMTs activity (Garro *et al*., 1991; Bielawski *et al*., 2002; Bonsch *et al*., 2006; Varela-Rey *et al*., 2013). We know that an exposure to alcohol during pregnancy can lead to abnormal brain development and cause fetal alcohol spectrum disorders (FASD), with symptoms ranging from craniofacial abnormalities to intellectual deficiency, behavioral difficulties and learning disabilities (Chudley *et al*., 2005; Cook *et al*., 2016; Legault *et al*., 2018). Although pioneer work demonstrated that early embryonic alcohol exposure can negatively influence development (Checiu and Sandor, 1986; Fazakas-Todea *et al*., 1986; Wiebold and Becker, 1987; Padmanabhan and Hameed, 1988), we still don’t fully understand how alcohol directly impacts the early embryo, especially its epigenome. A recent report shows that porcine zygotes exposed to alcohol *in vitro* have a lower rate of blastocyst formation, with blastocysts having increased mitochondrial dysfunctions and abnormal gene expression (Page-Lariviere *et al*., 2017). Haycock and Ramsay (2009) did show in a mouse model that alcohol exposure at E1.5 and E2.5 was associated with loss of *H19* imprinted DNA methylation in the placenta at E10.5 and growth restriction (Haycock and Ramsay, 2009). In early stage embryos, ethanol exposure seems to have a lasting impact on *Dnmt1* by reducing its expression, whereas *Dnmt3a* and *Dnmt3b* expression levels remained the same (Dasmahapatra and Khan, 2015). Since *Dnmt1* is required for the maintenance of DNA methylation profiles, especially imprinted gene methylation during the early embryonic reprogramming wave (Hirasawa *et al*., 2008; McGraw *et al*., 2013), alcohol exposure might compromise proper *Dnmt1* function and lead to altered epigenetic phenotypes. Although prenatal cigarette and recreational drugs (e.g., cocaine, cannabis) exposure have been linked to lasting behavioral and neurodevelopmental impairments, low birth weight, preterm birth, poor intrauterine growth and even infant death (Wehby *et al*., 2011), as well as alterations in DNA methylation and epigenetic profiles (Novikova *et al*., 2008; Toro *et al*., 2008; Breton *et al*., 2009; Guerrero-Preston *et al*., 2010; Suter *et al*., 2010; Toledo-Rodriguez *et al*., 2010; DiNieri *et al*., 2011), none of the prenatal expositions were done on pre-implantation embryos. As such, preclinical animal models of early embryonic exposure are needed to determine the deleterious consequence of cigarette smoking and recreational drugs on development, epigenome and gene expression. By being aware of the deleterious effects of cigarette and drug expositions onthe embryo during the first days of gestation days, women wanting to conceive might stop their consumption as preventive measures to protect their embryo.

Livestock does not have comparable environmental stressors, however, they are exposed to changing environmental conditions that affect their fertility. For example, livestock fertility, especially in dairy cows, is particularly vulnerable to higher temperature and humidity. Heat stress disrupts many metabolism processes (e.g., microtubules and microfilaments reorganization, reactive oxygen species production, DNA fragmentation and apoptosis) in embryos, leading to disrupted embryo development and increased embryonic mortality (Zhu *et al*., 2008; Koyama *et al*., 2012; de Barros and Paula-Lopes, 2018). Heat stress has a greater impact on pre-implantation embryos since heat resistance mechanisms are not fully developed at this stage. Embryos at 2-cell or 4-cell stage will be more affected since the acquisition of these processes overlaps with zygotic genome activation (ZGA) and early embryos do not respond to proapoptotic signals (de Barros and Paula-Lopes, 2018). One study suggests that the epigenetic changes seem to predominantly impact paternal imprinting genes, as the paternal genome is demethylated faster in the first days of embryo development compared to the maternal genome (Zhu *et al*., 2008). They reported that blastocysts resulting from mouse zygotes exposed to a 1 hour 40°C heat shock prior to IVC, showed loss of DNA methylation for paternally imprinted genes *H19* and *Igf*-*2r*, but normal DNA methylation for maternally imprinted genes *Peg1* and *Peg3*. However, since these embryos were only treated and cultured *in vitro*, it would be pertinent to retrieve oocytes, zygotes or embryos from livestock animals exposed to heat stress to define how genome-wide DNA methylation profiles are disturbed.

## Conclusion

Epigenetic modifications, specifically DNA methylation, play a crucial role in embryo development and are vulnerable to prenatal environmental factors and exposures occurring during the pre-implantation period. So far, a handful of *in vitro* studies have explored the effects of assisted reproductive technologies as well as prenatal environmental conditions and exposures, such as alcohol consumption and heat stress, during pre-implantation looking at short-term effects of severe epigenetic disturbances causing early manifestation of serious developmental phenotypes. Though mild impacts during pre-implantation are hugely understudied and may cause latent long-term effects on postnatal development. Therefore, there is a dire need to study the impacts of early embryo *in vitro* exposures past the blastocyst-stage using embryo transfer experiments, as well as early embryo *in vivo* exposure models, while also taking into consideration the importance of sex-specific variations and timing of exposure. Moreover, very few studies have been able to establish the direct link between DNA methylation alterations and observed phenotypes, mainly because of the limitations in studying the methylome in the early stages of development. Cutting-edge adaptations of standard whole-genome and reduced bisulfite genome sequencing technologies are now rapidly emerging, permitting high-resolution low-input single-cell methylation analyses. Thanks to these technological and intellectual advancements, as well as the integrative analysis of multi-omics layers, a promising future lies ahead for the study of pre-implantation epigenetics.
